# Impact of a modified produce prescription program on fruit and vegetable intake in an HBCU

**DOI:** 10.1371/journal.pone.0354520

**Published:** 2026-07-28

**Authors:** Janet Antwi, Redeemer Kofi Agbolegbe, Obed Akwaa Harrison, Kenneth Ofori-Panyin, Prince Dunyo, Mercedes Udodirim Guimaraes, Nora Bosio Onyeani

**Affiliations:** Department of Agriculture, Nutrition and Human Ecology, College of Agriculture, Food and Natural Resources, Prairie View A&M University, Prairie View, Texas, United States of America; TIU: Tishk International University, IRAQ

## Abstract

**Background:**

Limited access to fruits and vegetables remains a significant barrier among underserved populations, particularly within Historically Black Colleges and Universities (HBCUs). Evidence from previous studies suggests that prescription programs, which provide incentives for fresh produce consumption, are effective in enhancing dietary quality and alleviating food insecurity.

**Objective:**

This pilot investigation assessed the impact of a tailored produce prescription intervention on changes in fruit and vegetable consumption among students at a Historically Black College and University (HBCU).

**Methods:**

Fifty-five students were assigned to an intervention (n = 28) or control group (n = 27); both received nutrition education and cooking demonstrations, while the intervention group additionally received biweekly personalized produce bags. Fruit and vegetable intake was assessed using a 10-item dietary screener, supported by 24-hour recalls and food frequency questionnaires. Changes in intake were evaluated using descriptive statistics, chi-square tests, and Wilcoxon signed-rank tests.

**Results:**

Compared with the control group, the intervention group reported higher intake frequencies of cooked beans (χ^2^ = 17.63, p = 0.014), while the intake of non-deep-fried vegetables approached statistical significance (χ^2^ = 16.82, p = 0.052). Notably, only the intervention group experienced increases from baseline to endline in the intake of 100% fruit juice (Z = −2.199, p = 0.028), fruits (Z = −2.199, p = 0.028), green leafy vegetables (Z = −2.195, p = 0.028), cooked beans (Z = −2.776, p = 0.005), other non-deep-fried vegetables (Z = −2.292, p = 0.022), and tomato sauce (Z = −2.026, p = 0.043).

**Conclusion:**

The results of a modified produce prescription program revealed favorable within-group changes in the frequency of consumption of several fruit- and vegetable-related foods. While most between-group differences at endline were not statistically significant, observed patterns suggest potential promise and support the need for larger, adequately powered studies to evaluate the impact more rigorously.

## Introduction

Poor dietary habits, particularly low consumption of fruits and vegetables (F&V), remain a persistent public health concern in the United States, disproportionately affecting minority and low-income populations [1]. The Centers for Disease Control and Prevention (CDC) reports that only one in ten adults meets the daily recommended intake of fruits and vegetables, with even lower adherence observed among young adults, African Americans, and those living in food-insecure or underserved communities [2]. Students in Historically Black Colleges and Universities (HBCUs) often face structural barriers to accessing nutritious foods due to a lack of nutrition education, limited food outlets, and high prices of fresh produce, all of which contribute to poor dietary quality and increased risk for chronic conditions such as obesity, hypertension, and type 2 diabetes [3,4].

The development of strategies such as produce prescription (PRx) programs has attracted attention because of the potential of these programs to address food insecurity while promoting healthy eating. These interventions provide subsidized produce or vouchers to various individuals with diet-related health risks, linking food access with health outcomes [5]. Evidence suggests that PRx programs can increase F&V intake, improve diet quality, and support chronic condition management, particularly among underserved populations [6]. The majority of these programs, however, have been implemented in community or clinical health settings, with limited focus on collegiate residents, particularly students at HBCUs, who represent a distinct intersection of race, age, and socioeconomic susceptibility [7].

Despite the promising effects of PRx interventions in wider populations, little is known about their effectiveness in academic establishments, particularly in minority-serving environments [8]. Existing research on campus food environments shows that students often rely on calorie-dense, nutrient-poor choices because of affordability, convenience, or cultural preferences [9,10]. Similarly, there is a lack of research on modified or culturally tailored produce programs that account for the lived experiences and choices of Black college students, specifically in the context of behavioral and environmental change [11].

Given these gaps, this pilot study aimed to evaluate the effects of a modified produce prescription program on fruit and vegetable intake among students in Historically Black Colleges and Universities. We hypothesized that students who received the produce prescription intervention, in addition to nutrition education and cooking demonstrations, would demonstrate a greater increase in fruit and vegetable intake over 12 weeks compared to students who received education and demonstrations alone. This research is timely and necessary, given the growing interest in precision nutrition and culturally responsive interventions that address both dietary inequities and the social determinants of health in higher education settings.

## Methods

### Study design and setting

This pilot study used a 12-week pre–post quasi-experimental design to evaluate the effect of a modified produce prescription program on fruit and vegetable intake among food-insecure students. The study took place at a mid-sized Historically Black College and University (HBCU) in Texas, that serves a predominantly African American student population. The HBCU context is crucial because of the high prevalence of food insecurity documented in previous campus needs assessments and the presence of existing, although underutilized, food security resources [12]. Participants were assigned to either the intervention or control group using a non-randomized allocation approach. Group assignment was based on scheduling availability, willingness to participate in specific components of the intervention, and logistical considerations related to produce distribution. This pragmatic allocation strategy was chosen to accommodate the real-world constraints of implementing the intervention within a campus setting.

### Study population and eligibility criteria

Students were eligible if they were 18 years of age or older; self-identified as food insecure on the basis of responses to the USDA 6-item Food Security Module [13]; and/or were clinically diagnosed with prediabetes, diabetes, high blood pressure, and obesity/overweight; and were willing to attend nutrition education (NE) and cooking demonstration (CD) sessions during the 12-week program. Exclusion criteria included lack of access to food storage or preparation facilities for the entire study period or a medical condition that precluded consumption of fruits and vegetables. A total of 55 participants were enrolled after screening, and informed consent was provided. Recruitment strategies included targeted campus announcements, flyers placed in high-traffic areas, and email invitations through student listservs. Eligible students were assigned to an intervention group (n = 28) or a control group (n = 27).

### Intervention

The intervention group received a biweekly produce prescription bag for 12 consecutive weeks, from a central pick-up location on campus. Each bag contained a selection of fresh fruits and vegetables tailored to individual preferences and dietary goals, identified during a one-on-one session with the registered dietitian at the start of the program. Selections and serving sizes adhered to USDA MyPlate recommendations and emphasized variety, color, and nutrient density. The produce was sourced from local grocery stores to ensure freshness. To promote engagement, participants also received recipe cards, preparation tips, and short goal-setting prompts in their biweekly bags (S1 File). The intervention group additionally attended scheduled NE (three sessions) and five CD sessions delivered by a nutritionist. The sessions covered topics such as healthy meal planning, shopping on a budget, cooking with limited equipment, and food safety. Cooking demonstrations incorporated culturally relevant recipes using seasonal produce.

**Fig 1:**
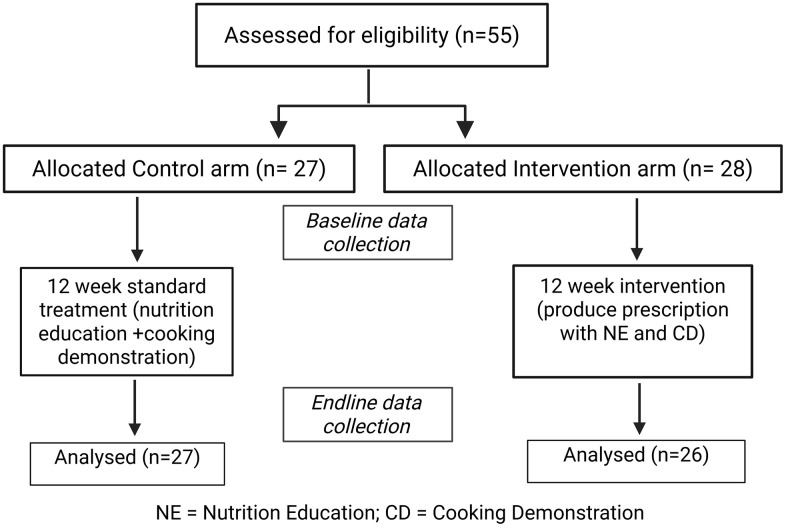
Methodology flowchart.

The control group received no produce prescriptions during the study but was invited to attend the same NE and CD sessions (Fig 1).

### Data collection

Baseline data and endline data included demographic information, self-reported food security status, and dietary intake. Attendance was recorded at all NE and CD sessions, and the distribution of produce bags was tracked biweekly for the intervention group. Recruitment and baseline data collection were conducted from February 5 to 23, 2024, and follow-up was completed in May 2024.

Fruit and vegetable intake was assessed using three complementary tools, including the Short 10-Item Dietary Assessment Tool [14], a validated instrument for assessing the frequency and portion sizes of usual fruit and vegetable consumption. 24-hour Dietary Recalls [15] – conducted at baseline and endline to capture detailed intake across two non-consecutive days (one weekday and one weekend day). Food Frequency Questionnaire (FFQ) [16] – used to assess longer-term consumption patterns and detect seasonal or habitual intake variations over the past month (30 days). Data collectors received standardized training to ensure consistency in administering dietary assessment tools. The primary outcome was a change in daily fruit and vegetable intake from baseline to 12 weeks. Fruit and vegetable intake was assessed using three complementary tools, namely the Short 10-Item Dietary Assessment Tool, 24-hour Dietary Recalls, and a Food Frequency Questionnaire (FFQ). For the analyses reported in this study, the primary outcome was derived from item-specific responses on the Short 10-Item Dietary Assessment Tool and presented as changes in the frequency of consumption of selected fruit- and vegetable-related food groups from baseline to 12 weeks.

### Data analysis

All the quantitative data were entered and analyzed using IBM SPSS Statistics version 28 (IBM Corp., Armonk, NY). Descriptive statistics were used to summarize baseline participant characteristics and program participation. Fruit and vegetable intake outcomes derived from the 10-item questionnaire were ordinal and measured repeatedly within individuals at baseline and endline using ordered frequency categories.

Between-group comparisons of categorical variables were conducted using Pearson’s chi-square tests to describe group differences at baseline and endline. Within-group changes in fruit and vegetable intake from baseline to endline were assessed using Wilcoxon signed-rank test, which is appropriate for paired ordinal data and does not assume normality or equal spacing between response categories. Given the quasi-experimental design, inference regarding intervention effects emphasized within-participant change rather than unadjusted between-group comparisons. No formal adjustment for multiple comparisons was applied; analyses were prespecified and exploratory, and the results are reported using unadjusted p-values and interpreted descriptively. Statistical significance was set at p < 0.05.

### Ethics statement

The study protocol received approval from the Prairie View A&M University Institutional Review Board. Written informed consent was obtained from all participants before enrollment. Participation was voluntary, and students could withdraw at any time without consequence. All the collected data were de-identified to maintain confidentiality, and all the study procedures adhered to the ethical principles outlined in the Declaration of Helsinki.

## Results

### Sociodemographic profile of the participants

The background characteristics of the study participants are shown in Table 1. There was a total of 55 participants: 27 in the control group and 28 in the intervention group. The participants had comparable characteristics. Most participants were females (approximately 78% in the control group and 82% in the intervention group). The mean age of the participants was 24.33 ± 6.58 years. Nearly all the participants (85%) were black/ African Americans and single. They were either not working (30%), working part-time (59%), working full-time, or on disability support (11%). Nearly half (48.5) lived off campus, while the rest resided on campus (40%) or with family (11%). More than half (52%) of the participants had a family history of type 2 diabetes.

**Table 1:** Background characteristics of the study participants at baseline.

Variables	Control (n = 27) n (%)	Intervention (n = 28) n (%)	Total (N = 55) n (%)	χ^2^	P-value^1^
**Gender**				0.184	.912
Male	5 (18.5)	4 (14.3)	9 (16.4)		
Female	21 (77.98)	23 (82.1)	44 (80.0)		
Nonbinary	1 (3.7)	1 (3.6)	2 (3.6)		
**Age (Years)** ^**1**^	25.4 (5.2)	23.3 (7.6)	24.3 (6.6)	0.206	.246
18–23	11 (40.7)	20 (71.4)	31 (56.4)	7.176	.066
24–29	13 (48.2)	6 (21.4)	19 (34.6)		
30–39	3 (11.1)	1 (3.6)	4 (7.3)		
≥ 40	0 (0.0)	1 (3.6)	1 (1.8)		
**Ethnicity**				0.840	.657
Black/African American	22 (81.5)	25 (89.3)	47 (85.5)		
Hispanic American	1 (3.7)	1 (3.6)	2 (3.6)		
Other	4 (14.8)	2 (7.1)	6 (10.9)		
**Classification**				*10.783*	***.029***
Freshman	0 (0.0)	5 (17.9)	5 (9.1)		
Sophomore	1 (3.7)	4 (14.3)	5 (9.1)		
Junior	4 (14.8)	7 (25.0)	11 (20.0)		
Senior	9 (33.3)	6 (21.4)	15 (27.3)		
Graduate	13 (48.2)	6 (21.4)	19 (34.6)		
**Marital Status**				0.003	.956
Single	23 (85.2)	24 (85.7)	47 (85.5)		
Married	4 (14.8)	4 (14.3)	8 (14.6)		
**Work Status**				4.610	.203
Not Working	8 (29.6)	16 (57.1)	24 (43.6)		
Part-time	16 (59.3)	9 (32.1)	25 (45.5)		
Full-time	2 (7.4)	2 (7.1)	4 (7.3)		
On Disability	1 (3.7)	1 (3.6)	2 (3.6)		
**Residence**				1.169	.558
Campus Hall	11 (40.7)	12 (42.9)	23 (41.8)		
Off campus	13 (48.2)	15 (53.6)	28 (51.0)		
With Family	3 (11.1)	1 (3.6)	4 (7.3)		
**Family History of T2D**				0.439	.508
Yes	14 (51.9)	17 (60.7)	31 (56.4)		
No	13 (48.2)	11 (39.3)	24 (43.6)		
**Food Security (FS)**				1.964	0.375
Very low FS	14 (51.9)	14 (50.0)	28 (50.9)		
Low FS	12 (44.4)	10 (35.7)	22 (40.0)		
High or marginal FS	1 (3.7)	4 (14.3)	5 (9.1)		

Significance determined by the independent t-test for continuous variables and the chi-square test for categorical variables; ^1^Mean (standard deviation). P-values <0.05 were considered significant.

### Intake of fruits and vegetables at baseline

**Fig 2:**
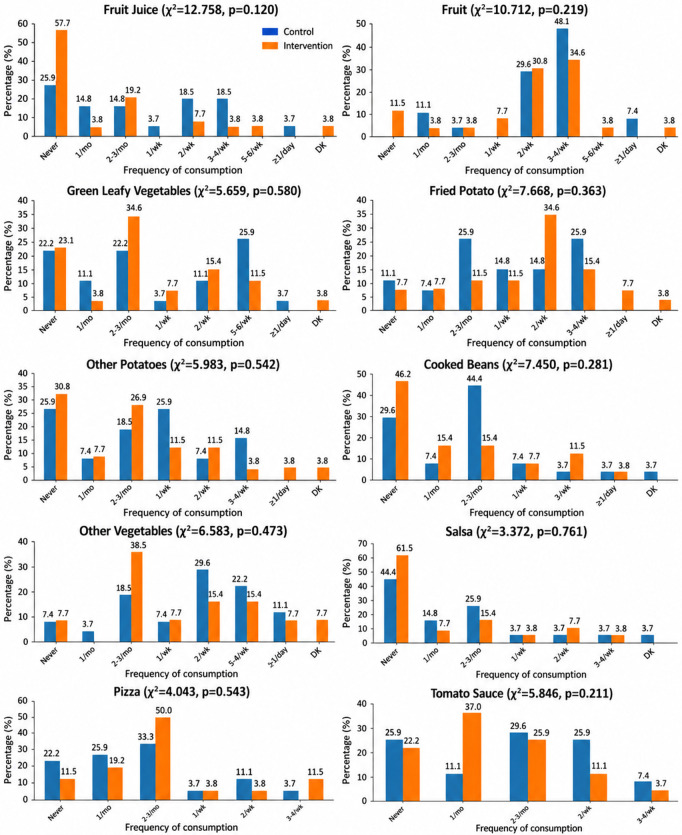
Fruit and vegetable consumption by study group at baseline.

Fig 2 presents baseline comparisons of the frequency of consumption of selected fruit- and vegetable-related food items between the control and intervention groups prior to the produce prescription intervention. Overall, no statistically significant between-group differences were observed across the 10 food categories shown (all p > 0.05), indicating that the two groups were broadly comparable in baseline dietary intake patterns. For fruit juice, a greater proportion of participants in the intervention group reported never consuming 100% fruit juice than in the control group (57.7% vs. 25.9%), although this difference was not statistically significant (χ^2^ = 12.758, p = 0.120). Fruit intake was similarly distributed across groups, with the highest proportions in both groups reporting consumption 2 or 3–4 times per week (χ^2^ = 10.712, p = 0.219). The prevalence of green leafy vegetables, fried potatoes, other potatoes, cooked beans, other vegetables, salsa, pizza, and tomato sauce also did not differ significantly across groups (all p > 0.05). Although modest variation in the distribution of some intake frequencies was evident, these differences were not statistically meaningful.

### Intake of fruits and vegetables at endline

**Fig 3:**
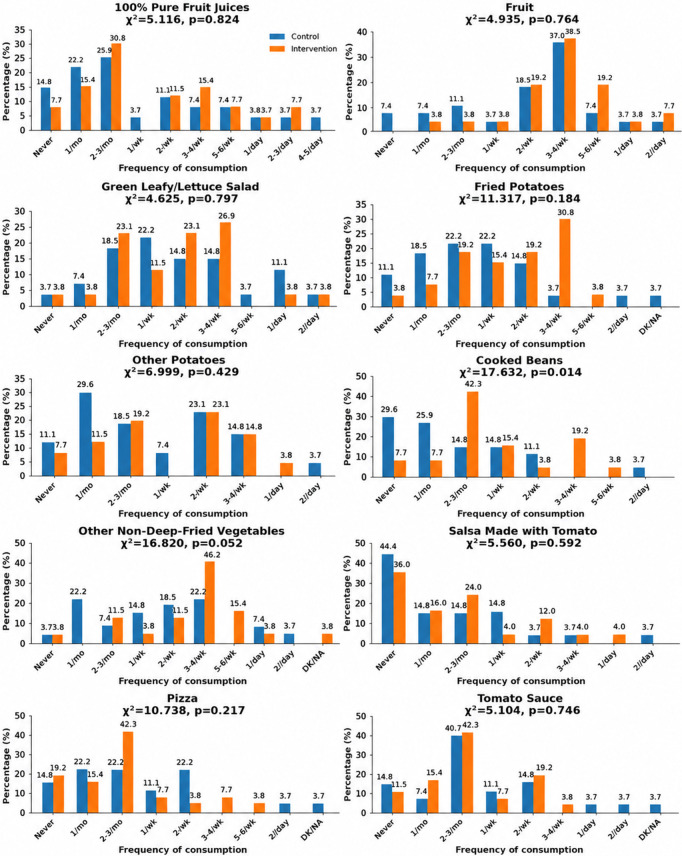
Fruit and vegetable consumption by study group at endline.

Fig 3 presents endline differences in the frequency of consumption across 10 fruit- and vegetable-related food groups between control (n = 27) and intervention (n = 26) participants. Overall, most between-group comparisons were not statistically significant. The only significant difference was observed for cooked bean consumption (χ^2^ = 17.632; p = 0.014), with intervention participants reporting higher intake frequencies than controls did. Specifically, compared with 14.8% of the control participants, 42.3% of the intervention participants consumed cooked beans 2–3 times per month, while 19.2% of the intervention group reported consuming beans 3–4 times per week, whereas none in the control group reported consuming beans.

Although the remaining food groups did not differ significantly, some differences in distribution were evident. Consumption of other vegetables that were not deep-fried approached statistical significance (χ^2^ = 16.820, p = 0.052), with 46.2% of the intervention participants reporting intake 3–4 times per week compared with 22.2% of the controls. Fried potato consumption also showed a noticeable difference in patterns, with 30.8% of the intervention participants reporting intake 3–4 times per week compared with 3.7% of the controls, although this difference was not significant (χ^2^ = 11.317, p = 0.184). For the remaining food groups, which included 100% fruit juice, fruit, green leafy salad, other potatoes, salsa, pizza, and tomato sauce, the frequency distributions were broadly similar across groups (all p > 0.05).

Table 2 shows within-group changes in the frequency of consumption of selected fruit- and vegetable-related food items from baseline (BL) to endline (EL) in the control and intervention groups. In the control group, a significant change was observed only for green leafy vegetables (Z = −2.256, p = 0.024). The proportion of participants who reported never consuming green leafy vegetables decreased from 6 (22.2%) at baseline to 1 (3.7%) at endline, while the proportion consuming them once per week increased from 1 (3.7%) to 6 (22.2%). No statistically significant changes were detected for fruit juice (Z = −0.158, p = 0.874), fruits (Z = −1.014, p = 0.310), fried potato (Z = −0.772, p = 0.444), other potatoes (Z = −0.188, p = 0.851), cooked beans (Z = −0.185, p = 0.853), other vegetables (Z = −1.587, p = 0.112), salsa (Z = −0.189, p = 0.850), pizza (Z = −1.587, p = 0.112), or tomato sauce (Z = −0.978, p = 0.328).

**Table 2:** Consumption of selected foods from baseline to endline.

Variable	Control (n = 27)		Z	P-Value	Intervention (n = 26)		Z	P-Value
	BL n (%)	EL n (%)			BL n (%)	EL n (%)		
**Fruit Juice (100%)**			−0.158	0.874			−2.199	**0.028**
***Never***	7 (25.9)	4 (14.8)			15 (57.7)	2 (7.7)		
***1/month***	4 (14.8)	6 (22.2)			1 (3.8)	4 (15.4)		
***2–3/month***	4 (14.8)	7 (25.9)			5 (19.2)	8 (30.8)		
***1/week***	1 (3.7)	1 (3.7)			0 (0.0)	0 (0.0)		
***2/week***	5 (18.5)	3(11.1)			2 (7.7)	3 (11.5)		
***3–4/week***	5 (18.5)	2 (7.4)			1 (3.8)	4 (15.4)		
***5–6/week***	0 (0.0)	2 (7.4)			1 (3.8)	2 (7.7)		
***1/day***	0 (0.0)	0 (0.0)			0 (0.0)	1 (3.8)		
***2–3/day***	1 (3.7)	1 (3.7)			0 (0.0)	2 (7.7)		
***4–5/day***	0 (0.0)	1 (3.7)			0 (0.0)	0 (0.0)		
***Don’t Know***	0 (0.0)	0 (0.0)			1 (3.8)	0 (0.0)		
**Fruits**			−1.014	0.310			**−2.199**	**0.028**
***Never***	0 (0.0)	2 (7.4)			3 (11.5)	0 (0.0)		
***1/month***	3 (11.1)	2 (7.4)			1 (3.8)	1 (3.8)		
***2–3/month***	1 (3.7)	3 (11.1)			1 (3.8)	1 (3.8)		
***1/week***	0 (0.0)	1 (3.7)			2 (7.7)	1 (3.8)		
***2/week***	8 (29.6)	5 (18.5)			8 (30.8)	5 (19.2)		
***3–4/week***	13 (48.1)	10 (37.0)			9 (34.6)	10 (38.5)		
***5–6/week***	0 (0.0)	2 (7.4)			1 (3.8)	5 (19.2)		
***1/day***	0 (0.0)	1 (3.7)			0 (0.0)	1 (3.8)		
***≥ 2/day***	2 (7.4)	1 (3.7)			0 (0.0)	2 (7.7)		
***Don’t Know***	0 (0.0)	0 (0.0)			1 (3.8)	0 (0.0)		
**Green leafy vegetables**			**−2.256**	**0.024**			−2.195	**0.028**
***Never***	6 (22.2)	1 (3.7)			6 (23.1)	1 (3.8)		
***1/month***	3 (11.1)	2 (7.4)			1 (3.8)	1 (3.8)		
***2–3/month***	6 (22.2)	5 (18.5)			9 (34.6)	6 (23.1)		
***1/week***	1 (3.7)	6 (22.2)			2 (7.7)	3 (1.5)		
***2/week***	3 (11.1)	4 (14.8)			4 (15.4)	6 (23.)		
***3–4/week***	7 (25.9)	4 (14.8)			3 (11.5)	7 (26.9)		
***5–6/week***	0 (0.0)	1 (3.7)			0 (0.0)	0 (0.0)		
***1/day***	1 (3.7)	3 (11.1)			0 (0.0)	1(3.8)		
***≥ 2/day***	0 (0.0)	1 (3.7)			1 (3.8)	1 (3.8)		
**Fried potato**			−0.772	0.444			−0.075	0.940
***Never***	3 (11.1)	3 (11.1)			2 (7.7)	1 (3.8)		
***1/month***	2 (7.4)	5 (18.5)			2 (7.7)	2 (7.7)		
***2–3/month***	7 (25.9)	6 (22.2)			3 (11.5)	5 (19.2)		
***1/week***	4 (14.8)	6 (22.2)			9 (34.6)	4 (15.4)		
***2/week***	4 (14.8)	4 (14.8)			4 (15.4)	5 (19.2)		
***3–4/week***	7 (25.9)	1 (3.7)			2 (7.7)	8 (30.8)		
***5–6/week***	0 (0.0)	0 (0.0)			0 (0.0)	1 (3.8)		
***≥ 2/day***	0 (0.0)	1 (3.7)			1 (3.8)	0 (0.0)		
***Don’t know***	0 (0.0)	1 (3.7)			0 (0.0)	0 (0.0)		
**Other potato**			−0.188	0.851			−1.651	0.099
***Never***	7 (25.9)	3 (11.1)			8 (30.8)	2 (7.7)		
***1/month***	2 (7.4)	8 (29.6)			2 (7.7)	3 (11.5)		
***2–3/month***	5 (18.5)	5 (18.5)			7 (26.9)	5 (19.2)		
***1/week***	7 (25.9)	2 (7.4)			3 (11.5)	6 (23.1)		
***2/week***	2 (7.4)	4 (14.8)			3 (11.5)	6 (23.1)		
***3–4/week***	4 (14.8)	4 (14.8)			1 (3.8)	3 (11.5)		
***5–6/week***	0 (0.0)	0 (0.0)			1 (3.8)	0 (0.0)		
***1/day***	0 (0.0)	0 (0.0)			1 (3.8)	1 (3.8)		
***≥ 2/day***	0 (0.0)	1 (3.7)			0 (0.0)	0 (0.0)		
**Cooked Beans**			−0.185	0.853			**−2.776**	**0.005**
***Never***	8 (29.6)	8 (29.6)			12 (46.2)	2 (7.7)		
***1/month***	2 (7.4)	7 (25.9)			4 (15.4)	2 (7.7)		
***2–3/month***	12 (44.4)	4 (14.8)			4 (15.4)	11 (42.3)		
***1/week***	2 (7.4)	4 (14.8)			2 (7.7)	4 (15.4)		
***2/week***	1 (3.7)	3 (11.1)			3 (11.5)	1 (3.8)		
***3–4/week***	1 (3.7)	0 (0.0)			1 (3.8)	5 (19.2)		
***5–6/week***	0 (0.0)	0 (0.0)			0 (0.0)	1 (3.8)		
***1/day***	1 (3.7)	0 (0.0)			0 (0.0)	0 (0.0)		
***≥ 2/day***	0 (0.0)	1 (3.7)			0 (0.0)	0 (0.0)		
**Other vegetables**			−1.587	0.112			**−2.292**	**0.022**
***Never***	2 (7.7)	1 (3.7)			2 (7.4)	1 (3.8)		
***1/month***	0 (0.0)	6 (22.2)			1 (3.7)	0 (0.0)		
***2–3/month***	10 (38.5)	2 (7.4)			5 (18.5)	3 (11.5)		
***1/week***	2 (7.7)	4 (14.8)			2 (7.4)	1 (3.8)		
***2/week***	4 (15.4)	5 (18.5)			8 (29.6)	3 (11.5)		
***3–4/week***	4 (15.4)	6 (22.2)			6 (22.2)	12 (46.2)		
***5–6/week***	2 (7.7)	0 (0.0)			3 (11.1)	4 (15.4)		
***1/day***	0 (0.0)	2 (7.4)			0 (0.0)	1 (3.8)		
***≥ 2/day***	0 (0.0)	1 (3.7)			0 (0.0)	0 (0.0)		
***Don’t know***	0 (0.0)	0 (0.0)			0 (0.0)	1 (3.8)		
**Salsa**			−0.189	0.850			−1.500	0.134
***Never***	12 (44.4)	12 (44.4)			16 (61.5)	9 (36.0)		
***1/month***	4 (14.8)	4 (14.8)			2 (7.7)	4 (16.0)		
***2–3/month***	7 (25.9)	4 (14.8)			4 (15.4)	6 (24.0)		
***1/week***	1 (3.7)	4 (14.8)			1 (3.8)	1 (4.0)		
***2/week***	1 (3.7)	1 (3.7)			2 (7.7)	3 (12.0)		
***3–4/week***	1 (3.7)	1 (3.7)			1 (3.8)	1 (4.0)		
***5–6/week***	1 (3.7)	0 (0.0)			0 (0.0)	0 (0.0)		
***1/day***	0 (0.0)	0 (0.0)			0 (0.0)	1 (4.0)		
***≥ 2/day***	0 (0.0)	1 (3.7)			0 (0.0)	0 (0.0)		
***Don’t Know***	0 (0.0)	0 (0.0)			0 (0.0)	0 (0.0)		
**Pizza**			−1.587	0.112			−0.081	0.935
***Never***	6 (22.2)	4 (14.8)			3 (11.5)	5 (19.2)		
***1/month***	7 (25.9)	6 (22.2)			5 (19.2)	4 15.4)		
***2–3/month***	9 (33.3)	6 (22.2)			13 (50.0)	11 (42.3)		
***1/week***	1 (3.7)	3 (11.1)			1 (3.8)	2 (7.7)		
***2/week***	3 (11.1)	6 (22.2)			1 (3.8)	1 (3.8)		
***3–4/week***	1 (3.7)	0 (0.0)			3 (11.5)	2 (7.7)		
***5–6/week***	0 (0.0)	0 (0.0)			0 (0.0)	1 (3.8)		
***≥ 2/day***	0 (0.0)	1 (3.7)			0 (0.0)	0 (0.0)		
***Don’t know***	0 (0.0)	1 (3.7)			0 (0.0)	0 (0.0)		
**Tomato Sauce**			−0.978	0.328			**−2.026**	**0.043**
***Never***	7 (25.9)	4 (14.8)			6 (22.2)	3 (11.5)		
***1/month***	3 (11.1)	2 (7.4)			10 (37.0)	4 (15.4)		
***2–3/month***	8 (29.6)	11 (40.7)			7 (25.9)	11 (42.3)		
***1/week***	0 (0.0)	3 (11.1)			0 (0.0)	2 (7.7)		
***2/week***	7 (25.9)	4 (14.8)			3 (11.1)	5 (19.2)		
***3–4/week***	2 (7.4)	0 (0.0)			1 (3.8)	1 (3.8)		
***1/day***	0 (0.0)	1 (3.7)			0 (0.0)	0 (0.0)		
***≥ 2/day***	0 (0.0)	1 (3.7)			0 (0.0)	0 (0.0)		
***Don’t know***	0 (0.0)	1 (3.7)			0 (0.0)	0 (0.0)		

Significance was defined as a P-value <0.05 for Wilcoxon signed-rank test.

In the intervention group, significant within-group changes were observed for fruit juice (Z = −2.199, p = 0.028), fruits (Z = −2.199, p = 0.028), green leafy vegetables (Z = −2.195, p = 0.028), cooked beans (Z = −2.776, p = 0.005), other vegetables (Z = −2.292, p = 0.022), and tomato sauce (Z = −2.026, p = 0.043). Fruit juice intake improved markedly, with the proportion reporting never consuming it decreasing from 15 (57.7%) to 2 (7.7%), and increasing in more frequent intake categories. Fruit consumption also improved, as never consumption declined from three (11.5%) to zero, while daily or higher intake emerged at endline. Green leafy vegetable intake increased, with never consumption falling from 6 (23.1%) to 1 (3.8%) and consumption at 3–4 times per week rising from 3 (11.5%) to 7 (26.9%). Cooked beans showed a notable shift, with never consumption decreasing from 12 (46.2%) to 2 (7.7%) and intake 2–3 times per month increasing from 4 (15.4%) to 11 (42.3%). The intake of other vegetables also improved, particularly in the 3–4 times per week category, which increased from 6 (22.2%) to 12 (46.2%). Tomato sauce intake shifted toward more regular use, with never consumption declining from 6 (22.2%) to 3 (11.5%) and 2–3 times per month increasing from 7 (25.9%) to 11 (42.3%). No significant changes were detected for fried potato (p = 0.940), other potatoes (p = 0.099), salsa (p = 0.134), or pizza (p = 0.935).

Overall, the results revealed that although the intervention group demonstrated several favorable within-group changes from baseline to endline, the control group also showed improvement in green leafy vegetable intake, and between-group differences at endline were largely non-significant except for those for cooked beans. Accordingly, these findings should be interpreted cautiously and do not permit the attribution of all observed changes solely to the produce prescription intervention process.

## Discussion

This 12-week pilot, quasi-experimental study examined whether participation in a tailored produce prescription program was associated with changes in fruit- and vegetable-related intake among students at HBCUs. Baseline intake patterns were broadly comparable between intervention and control participants across the ten food categories assessed. At endline, between-group comparisons revealed a significant difference in cooked bean intake frequencies, with higher consumption reported among intervention participants, while non-deep-fried vegetable intake showed a similar pattern but only approached statistical significance. Consistent with a within-participant focus, within-group analyses indicated that only the intervention group experienced significant improvements across several items (including 100% fruit juice, fruits, green leafy vegetables, cooked beans, other non-deep-fried vegetables, and tomato sauce), whereas the control group showed a significant change only for green leafy vegetables. These results suggest that modest, targeted dietary shifts may be feasible in a short-duration campus program; however, the limited number of statistically significant between-group differences likely reflects constraints in study power and intervention intensity and warrants closer consideration of context and mechanisms.

Evidence from larger and more intensive produce prescription programs provides useful context for the modest between-group differences observed in this pilot study. For example, Hager et al. conducted a multisite evaluation of nine produce prescription programs across the United States and reported significant increases in fruit and vegetable intake, along with improvements in food security and cardiometabolic health [17]. In contrast, the present pilot study was limited by a relatively short duration, a modest “dose” of produce support, and the concurrent exposure of both groups to nutrition education and cooking demonstrations, components that may have improved awareness and behaviors in both arms and reduced between-group separation. In addition, the small sample size limited the statistical power to detect modest shifts across multiple food categories, even when distributional changes were directionally favorable [17].

Other program evaluations emphasize the importance of sustained exposure and reinforcement. In Washington State, Marcinkevage et al. reported that a $10 fruit and vegetable voucher program achieved high redemption rates, and most participants reported increasing their consumption as a result [18]. This aligns with our finding that beans, a culturally familiar, affordable, and versatile food, were more readily adopted than other produce items. In pediatric contexts, Ridberg et al. observed that produce prescriptions improved both household food security and children’s fruit and vegetable intake, but these interventions often included parental engagement and structured support, which may have reinforced dietary changes [5].

The college setting introduces unique challenges to dietary change that likely influenced our results. Nikolaus et al. and Nazmi et al. reported that food environments, time constraints, cooking facilities, and competing priorities can limit students’ ability to consistently integrate fresh produce into their diets [19,20]. Additionally, unmeasured factors such as taste preferences, perceived self-efficacy for meal preparation, social support, cooking skills, and the campus food environment (including reliance on convenience foods or dining options) may mediate uptake [17]. Without more intensive behavioral support, such as cooking demonstrations tied to distributed produce or regular follow-up on goal attainment, students may default to existing eating habits, especially if they already consume certain vegetables at baseline. This ceiling effect, noted by Hager et al., can limit the measurable impact of short-term interventions [17]. By removing preparation barriers entirely, Berkowitz et al. demonstrated that more structured food provision models, such as medically tailored meals, can produce larger dietary changes [21]. While our model emphasized choice and cultural tailoring, it still relied on students preparing the produce themselves, which may have constrained the uptake of certain items.

The modest and sometimes borderline between-group differences observed in this pilot likely reflect a combination of limited intervention intensity, short follow-up, shared education exposure across groups, and campus-specific environmental barriers [17,22]. Nonetheless, the observed improvement in cooked bean intake suggests that emphasizing culturally familiar, practical foods may be a productive entry point for Food-is-Medicine strategies in HBCU settings [23]. From an implementation perspective, campus-based produce prescription programs may be strengthened by (i) aligning distributed items with low-equipment, time-efficient recipes; (ii) embedding structured reinforcement (brief check-ins, goal tracking, peer ambassadors, or digital reminders); (iii) partnering with dining services, campus pantries, or mobile markets to increase convenience and reduce stigma; and (iv) testing delivery versus pickup models to improve utilization. Future studies should be adequately powered, track intervention “dose” and produce utilization, and incorporate qualitative methods to identify mechanisms and optimize program design for food-insecure students in under-resourced campus environments.

### Strengths and limitations

A key strength of this study lies in its specific focus on fruit and vegetable intake changes in response to a produce prescription program within an HBCU, an underrepresented context in the literature. The intervention’s individualized produce selections, guided by a registered dietitian and aligned with USDA MyPlate recommendations, ensured cultural and contextual relevance. Incorporating nutrition education and goal setting likely contributed to the uptake of certain items, and the use of multiple dietary assessment tools strengthened the validity of our measures.

However, several limitations help explain the limited scope of change. The 12-week duration and fixed produce quantity may have been insufficient to shift ingrained eating habits, particularly compared to longer, higher-dosage programs that have shown stronger results [17,18]. The absence of structured reinforcement tied directly to the weekly produce, such as live cooking demonstrations or recipes requiring minimal preparation, may have limited the integration of the produce into daily diets. Baseline consumption for some items was already relatively high which may be due to unintentional sensitization of the population to healthy eating during needs assessment prior to the intervention, resulting in less room for measurable improvement, a phenomenon observed in other produce prescription research [17]. Environmental constraints typical of college settings, including irregular schedules and limited cooking facilities, likely reduced the potential for impact, which is consistent with the findings of Sogari et al., who, using an ecological model, demonstrated that transitions to independent living, unpredictable routines, and restricted cooking resources substantially hinder healthy food consumption, particularly fruits and vegetables, among college-aged individuals [24]. Finally, the small sample size limits the generalizability of our findings, reduces statistical power, and constrains the use of regression-based models, meaning that only the most pronounced, item-specific changes, such as beans, reached significance which aligns with the work of Zhang et al., who emphasized that dietary intervention studies targeting episodically consumed foods, such as vegetables and beans, require careful power and sample size planning, as insufficient sample sizes can greatly reduce the ability to detect meaningful intervention effects in these contexts [25]. Given multiple item-level tests, the findings should be interpreted cautiously and viewed as hypothesis-generating.

Despite these constraints, the trends observed in this study align with the broader literature, indicating that culturally tailored produce prescriptions may influence dietary behavior. Scaling program duration, increasing produce allotment, and embedding stronger behavioral support may yield more substantial and consistent improvements in fruit and vegetable intake among HBCU students.

## Conclusion

This pilot study examined the feasibility of a produce prescription program for supporting fruit and vegetable consumption among college students at a Historically Black College and University. Modest dietary changes were observed, with within-group analyses indicating improvements across several fruit- and vegetable-related food items among intervention participants. While most between-group differences at endline were not statistically significant, descriptive patterns suggested higher intake of cooked beans and non-deep-fried vegetables in the intervention group. Baseline intake patterns were broadly comparable between groups, supporting internal validity. Collectively, these findings suggest that the development of produce prescription interventions may be feasible and show potential to support dietary improvement in food-insecure college populations, while underscoring the need for well-controlled, larger, adequately powered studies with longer durations and enhanced behavioral support to evaluate the impact more rigorously.

## Supporting information

S1 File Produce prescription dataset. (XLSX)
